# The Effectiveness of Text Support for Stopping Smoking in Pregnancy (MiQuit): Multi-Trial Pooled Analysis Investigating Effect Moderators and Mechanisms of Action

**DOI:** 10.1093/ntr/ntae026

**Published:** 2024-02-14

**Authors:** Joanne Emery, Jo Leonardi-Bee, Tim Coleman, Lisa McDaid, Felix Naughton

**Affiliations:** School of Health Sciences, University of East Anglia, Norwich, UK; Centre for Evidence Based Healthcare, University of Nottingham, Nottingham, UK; Division of Primary Care, University of Nottingham, Nottingham, UK; School of Health Sciences, University of East Anglia, Norwich, UK; School of Health Sciences, University of East Anglia, Norwich, UK

## Abstract

**Introduction:**

Digital cessation support appeals to pregnant smokers. In two pooled RCTs, MiQuit, a pregnancy-specific tailored text messaging intervention, did not show effectiveness for validated prolonged abstinence. However, secondary outcomes and potential moderators and mediators have not been investigated. We aimed to determine, using pooled RCT data: (1) MiQuit effectiveness on a range of smoking outcomes; (2) whether baseline tobacco dependence or quit motivation moderate effectiveness; (3) whether hypothesized mechanisms of action (quitting determination, self-efficacy, baby harm beliefs, lapse prevention strategies) mediate effectiveness.

**Methods:**

Pooled data analysis from two procedurally identical RCTs comparing MiQuit (*N* = 704) to usual care (*N* = 705). Participants were smokers, <25 weeks pregnant, recruited from 40 English antenatal clinics. Outcomes included self-reported 7-day abstinence at 4 weeks post-baseline and late pregnancy, and prolonged abstinence. Late pregnancy outcomes were also biochemically validated. We used hierarchical regression and structural equation modeling.

**Results:**

MiQuit increased self-reported, 7-day abstinence at 4 weeks (OR = 1.73 [95% CI 1.10–2.74]) and was borderline significant at late pregnancy (OR = 1.34 [0.99–1.82]) but not for prolonged or validated outcomes. Effectiveness was not moderated by baseline dependence (heaviness of smoking “low” vs. “moderate–high”) or motivation (planning to quit ≤30 days [high] vs. >30 days [low]), but effects on self-reported outcomes were larger for the high motivation sub-group. MiQuit had a small effect on mean lapse prevention strategies (MiQuit 8.6 [SE 0.17], UC 8.1 [SE 0.17]; *P* = .030) but not other mechanisms.

**Conclusions:**

MiQuit increased short-term but not prolonged or validated abstinence and may be most effective for those motivated to quit sooner.

**Implications:**

Digital cessation support appeals to pregnant smokers. MiQuit, a tailored, theory-guided text messaging program for quitting smoking in pregnancy, has not shown effectiveness for validated prolonged abstinence in two previous RCTs but its impact on other smoking outcomes and potential mechanisms of action are unknown. When pooling trial data, MiQuit increased self-reported short-term abstinence, including making a quit attempt and abstinence at 4-week follow-up, but not late pregnancy, sustained, or validated abstinence. MiQuit appeared effective at late pregnancy for participants with high quitting motivation, but its mechanisms of action remain uncertain. Additional support components are likely required to enhance effectiveness.

## Introduction

Smoking in pregnancy (SIP) is an international public health problem and a leading cause of poor pregnancy and infant health outcomes, such as prematurity, low gestational weight, stillbirth, and sudden infant death.^1–3^ SIP disproportionately affects people from lower socioeconomic groups, perpetuating health inequalities.^4–6^ Over half of smokers try to quit during pregnancy,^7^ but most quit attempts go unaided,^8^ greatly reducing their chances of success.^9^ In England, National Health Service (NHS) support for stopping SIP, consisting of interpersonal counseling and the offer of nicotine replacement therapy (NRT), is highly effective^9^ but uptake is low.^8,10^

Digital self-help cessation support, such as support delivered by computer or smartphone, is attractive to pregnant smokers,^11^ and review evidence shows it can be effective for this group.^12^ Effective support for quitting SIP needs to be pregnancy-specific,^13^ reflecting key cessation motivators for this group such as the desire to protect the baby and a limited time frame.^7^ Tailored support, ie, support content that is adapted to user characteristics/contexts including smoking beliefs, behavior, and demographics, appears more effective than non-tailored/generic support^14^; possibly due to increased salience of information perceived as personally relevant.^15^

MiQuit, a low-cost, tailored, theory-guided, 12-week text messaging programme for quitting SIP, shows high acceptability, delivery fidelity, and engagement among pregnant women who smoke.^16–18^ However, despite promising findings from feasibility and pilot randomized controlled trials (RCTs),^16,18^ a trial sequential analysis pooling the pilot trial with a third large RCT, which was procedurally identical, did not find evidence that MiQuit increased biochemically-validated prolonged abstinence between 4 weeks post-baseline and late pregnancy (around 15 weeks’ gestation to around 36 weeks’ gestation).^19^ In these pooled trials, only two-thirds of participants who self-reported abstinence engaged in biochemical verification, therefore reducing statistical power, and it is unknown whether MiQuit may be effective for self-reported and other secondary smoking outcomes, for which more data was collected. While prolonged abstinence is the preferable outcome for smoking cessation in pregnancy, shorter periods of abstinence may still provide a benefit for the fetus given the dose-response relationship between smoking and some pregnancy complications.^3^

MiQuit texts are grouped into component message types each targeting a key determinant of quitting smoking in pregnancy: motivation to quit (messages targeting reasons for quitting and outcome expectancies), self-efficacy to quit (messages aiming to increase self-efficacy), baby harm beliefs (messages about risks), and use of lapse prevention strategies (messages targeting relapse prevention).^16^ Research, however, has yet to explore whether MiQuit’s message types affect their target determinants and potentially drive changes in smoking behavior. Also, as a low-intensity intervention, it is possible that high tobacco dependence or low quit motivation, which are negatively associated with achieving smoking abstinence in pregnancy,^20^ may diminish the benefits from MiQuit and require additional content or components to address.

In this study, using pooled data from two large MiQuit RCTs with identical procedures, we aim to determine: (1) the effect of MiQuit for a range of smoking outcomes; (2) whether baseline tobacco dependence and quit motivation moderate MiQuit effectiveness; (3) whether hypothesized MiQuit mechanisms of action (quitting determination, quitting self-efficacy, baby harm beliefs, number of lapse prevention strategies used) mediate the intervention effect.

## Methods

### Design, Participants, and Randomization

This was a secondary analysis of two large, combined, procedurally identical RCTs with individually randomized, multicenter, parallel-group designs.^18,19^ Participants were recruited from 40 English NHS hospital antenatal clinics between February and September 2014^18^ and between December 2017 and February 2019.^19^ Consent was obtained in clinic or verbally by telephone. Further details of recruitment procedures are reported in the study protocols.^21,22^ Participants were <25 weeks’ gestation, aged 16 years or over, smoked at least five daily cigarettes pre-pregnancy and at least one currently, and not already receiving text support for smoking cessation. They were not necessarily motivated to quit smoking but were willing to accept information about smoking cessation. Participants were randomized, following baseline data collection, in a 1:1 ratio using computer-generated blocks of randomly varying size.

### Treatments and Study Procedures

#### Usual Care (UC)

Participants received a generic (non-tailored) NHS booklet on quitting smoking in pregnancy and were free to use any cessation support available within usual NHS antenatal care.

#### MiQuit Intervention

Participants received UC plus the 12-week MiQuit programme of tailored self-help text messages. Full details of MiQuit are published elsewhere, which include examples of tailored messages.^16,17^ MiQuit texts are personalized by name and gestation and tailored to 12 baseline participant characteristics such as tobacco dependence, quit motivation, and smoking trigger situations. MiQuit is intended for use by both more and less motivated quitters; it aims to encourage quit attempts in those unmotivated to quit and to support abstinence in those who make a quit attempt. MiQuit does not currently provide advice on using NRT or facilitate access to it.

#### Procedures

At baseline, demographic and smoking data were collected by telephone survey, including determination and self-efficacy to quit, and baby harm from smoking beliefs. Four weeks after randomization (“4-week follow-up”), a researcher phoned participants to ask about smoking in the previous week. At 36 weeks’ gestation (“late pregnancy follow-up”), a researcher phoned again to ask about quit attempts, smoking in the previous week, smoking since the 4-week contact, and use of lapse prevention strategies. If, after several attempts, phone call contact was unsuccessful, we posted and e‐mailed a link to the questionnaire. If participants reported total abstinence (“not even a puff”) for the previous week at late pregnancy, a visit or remote collection pack was arranged to collect exhaled-breath carbon monoxide (CO) readings and/or saliva samples for biochemical validation. All participants were offered £5 in shopping vouchers for completing data collection at each contact (baseline, 4 weeks, and late pregnancy). In the second trial,^19^ an additional £10 was offered if data were provided for all three contacts (maximum £45). A £10 voucher (£30 in the second trial) was offered for providing a CO or saliva sample among participants who self-reported 7-day abstinence at late pregnancy, regardless of the result of this. Full details of procedures can be found elsewhere.^18.19,21,22^

## Measures

### Smoking Outcomes

We analyzed, for the pooled data, the seven smoking abstinence outcomes reported in the two MiQuit RCTs.^18,19^ These included self-reported outcomes (7-day point prevalence abstinence [“not even a puff”] (i) at 4 weeks post-baseline; (ii) at late pregnancy; (iii) at both follow-ups; and (iv) prolonged abstinence [no more than five cigarettes in total] between both follow-ups), and outcomes with biochemical validation (7-day point prevalence abstinence (v) at late pregnancy; (vi) at both follow-ups [validated only at late pregnancy]; and (vii) prolonged abstinence between both follow-ups [validated only at late pregnancy]). Additionally, we used the self-reported number of quit attempts reported at late pregnancy (since baseline and lasting at least 24 h) to categorize participants as (viii) having made at least one quit attempt or not. A small number of participants who self-reported achieving a smoking abstinence outcome but who reported no quit attempt or were missing quit attempt data (*N* = 12; 8 UC, 4 MiQuit) were re-classified as having made a quit attempt, as all participants were smoking at baseline. All eight outcomes are binary. As MiQuit aims to promote abstinence, smoking reduction was not investigated as an outcome.

### Hypothesized Moderator Variables

We explored the effect of two hypothesized moderator variables: baseline tobacco dependence and baseline quit motivation. Heaviness of Smoking Index (HSI),^23^ calculated from the baseline number of daily cigarettes and time to first cigarette, was used to categorize participants as low dependence (HSI 0–2) or moderate to high dependence (HSI 3–6). A single baseline intention-to-quit item^24^ was used to categorize participants as high motivation (planning to quit within the next 2 weeks or within the next 30 days) or as low motivation (planning to quit within the next 3 months or not planning to quit). Both measures predict smoking cessation behaviors in pregnancy,^20,25–27^ and emerged as the only significant predictors among baseline demographic, cognitive, and behavioral variables in multivariate models,^26^ so are the focus as potential moderators here.

### Hypothesized Mediator Variables

We explored the effect of four hypothesized mediator variables; three related to quitting beliefs that predict smoking cessation behaviors in pregnancy^26^ (determination to quit, self-efficacy to quit, baby harm beliefs), and number of lapse prevention strategies used.^28^ The three quitting beliefs were measured at both baseline and late pregnancy, on five-point scales from “not at all” (1) to “extremely” (5), and changes in scores between time points were calculated (potential range –4 to 4). Determination to quit was measured by a single item that asks “How determined are you to stop smoking until your baby is born?.”^16^ Self-efficacy was measured by a four-item scale (*α* = .81), developed for use with pregnant women who smoke,^16^ that asks “How confident are you that you can stop smoking until your baby is born?,” and “How confident are you that you can avoid smoking (after a meal/with other smokers/ when anxious or stressed)?” Self-efficacy scores represent the mean of the four items. Baby harm beliefs were measured by a single item that asks “How much do you agree with the statement: ‘Smoking during pregnancy can cause serious harm to my baby’?.”^16^ Number of lapse prevention strategies used since baseline, self-reported at late pregnancy only, was the sum of cognitive or behavioral lapse prevention strategies used, at least once, out of a possible 15 strategies listed.^28^

### Data Analysis

We followed a pre-specified protocol. Analyses were conducted in Stata v16, using the pooled data from the two trials and conducted using the intention to treat principle. All statistical tests were two-tailed and assessed at the 5% significance level. Outcome data were coded as “smoking” for all participants who were missing smoking outcomes (Russell Standard, considered a gold standard for the measurement of smoking cessation outcomes),^29^ other than making a quit attempt, where a complete case analysis was used. We used the generalized structural equation modeling (“GSEM”) procedure for mediation analyses as outcomes were binary and the data structure hierarchical; indirect and total effect estimates were obtained using the “medeff” post-estimation command.^30^

### Sample Size and Data Attrition

The original trial papers give details of sample size calculations^18,19^; there were *N* = 407 and *N* = 1002 participants in trials 1 and 2, respectively. Of 1409 participants in the pooled data at baseline (MiQuit *N* = 704; UC *N* = 705), 73% (*N* = 1033) provided smoking outcome data at the 4 week follow-up (MiQuit 70% [*N* = 492]; UC 77% [*N* = 541]), and 64% (*N* = 907) provided smoking outcome data at the late pregnancy follow-up (MiQuit 62% [*N* = 438]; UC 67% [*N* = 469]). Quit attempt data at the late pregnancy follow-up was provided by 63% (*N* = 884) of baseline participants (MiQuit 61% [*N* = 429]; UC 65% [*N* = 455]). Of those who self-reported abstinence at late pregnancy (*N* = 199), 66% (*N* = 132) underwent biochemical validation (MiQuit 66% [*N* = 74]; UC 67% [*N* = 58]).

### Smoking Outcomes

We calculated the frequencies and percentages of participants, within each treatment arm, achieving each of the eight smoking outcomes. Hierarchical logistic regression models, with adjustment for trial as a random intercept, were used to estimate the odds ratio (OR), with 95% confidence intervals (CI), for the effect of MiQuit on each of the eight smoking outcomes.

### Moderator Analyses

We calculated frequencies and percentages and used hierarchical logistic regression models as above, but within each of two levels of the dichotomized moderator variable. The effect of the hypothesized moderator was assessed by including a fixed effect interaction term between the treatment arm and the dichotomous moderator variable within a hierarchical model that included both levels of the moderator.

### Mediator Analyses

We explored mediation by analyzing (i) between-arm differences in hypothesized mechanisms of MiQuit action and (ii) mediation of MiQuit effectiveness via these mechanisms. The four hypothesized mechanisms of action (mediators) were treated as continuous variables.

1. Hierarchical linear regression models, with adjustment for trial as a random intercept, were used to investigate between-arm differences in four hypothesized mechanisms of MiQuit action measured at late pregnancy. For the three quitting beliefs, we controlled for participants’ baseline level of the same variable by including this as a fixed effect within the hierarchical model. A complete case analysis was used for all outcomes.
2. Mediation analysis was carried out using structural equation modelling (SEM) to measure the direct, indirect, and total effects of MiQuit on smoking abstinence via pathways through the four potential mediators (hypothesized mechanisms of action above). Hierarchical logistic mediation models, with adjustment for trial as a random intercept, were used to estimate model parameters. Indirect (mediation) effects were tested for when the conditions of mediation were met ie, evidence of both a significant effect of MiQuit on the potential mediator and a significant effect of the potential mediator on the smoking outcome. Given the causal logic of mediation analysis, smoking outcomes were restricted to those measured at late pregnancy. Self-reported 7-day abstinence at late pregnancy was pre-specified as our primary SEM outcome to maximize information size and, therefore, statistical power; we used the biochemically validated outcome in a sensitivity analysis. SEM analyses were carried out on complete case smoking outcomes as mediators and smoking outcomes were missing concurrently (both were measured at late pregnancy follow-up).

In SEM terminology, the treatment arm and trial comprised our exogenous (independent) variables; potential mediators and smoking outcomes comprised our endogenous (dependent) variables. All endogenous variables were observed, ie, had measured values. Correlations between potential mediator variables, where significant, were included in the models. Akaike’s information criterion (AIC) and Bayesian information criterion (BIC) were used to assess model fit.

## Results

### Participants

Characteristics were similar between treatment arms. Table 1 shows baseline descriptive statistics, per arm, for the pooled trial data. Participants (*N* = 1409) were, on average, 27 years old and 15 weeks pregnant; 94% were of White ethnicity, 70% had no post-16 qualifications (those taken beyond the compulsory UK schooling ages of 5–16), 64% had a partner who smoked and 67% were not in their first pregnancy. Baseline tobacco dependence was classed as low (HSI 0–2) in 62% of participants and as moderate to high (HSI 3–6) in 38% of participants. Baseline quit motivation was classed as low (not planning to quit within the next 30 days) in 46% of participants and as high (planning to quit within the next 30 days) in 54% of participants.

**Table 1.** Key Baseline Characteristics Per Treatment Arm For the Combined MiQuit RCTs

Characteristic	Usual Care (*N* = 705)	MiQuit (*N* = 704)
Age		
Mean (SD)	27.2 (5.7)	26.9 (5.7)
Median (Q1, Q3)	26.6 (22.8, 30.9)	26.3 (22.6, 31.0)
Min, max	16.4, 43.2	16.7, 43.4
Ethnicity		
White	661 (93.8)	657 (93.3)
Mixed race	27 (3.8)	24 (3.4)
Other	15 (2.1)	20 (2.8)
Missing	2 (0.3)	3 (0.4)
Education		
No qualifications	120 (17.0)	115 (16.3)
GCSEs/equivalent	371 (52.6)	383 (54.4)
A levels/equivalent	146 (20.7)	148 (21.0)
Degree or higher	59 (8.4)	53 (7.5)
Missing	9 (1.3)	5 (0.7)
Gestation in weeks Mean (SD)	15.1 (4.0)	15.0 (4.0)
Median (Q1, Q3)	13.4 (12.3, 19.7)	13.3 (12.3, 19.6)
Min, max	3.9, 24.9	6.0, 24.7
Previous pregnancies beyond 24 weeks		
None	227 (32.2)	243 (34.5)
One or more	478 (67.8)	461 (65.5)
Partner smoking status		
Single	113 (16.0)	119 (16.9)
Partner a non-smoker	147 (20.9)	124 (17.6)
Partner a smoker	445 (63.1)	461 (65.5)
Longest previous quit attempt Quit not attempted	157 (22.3)	176 (25.0)
<2 weeks	163 (23.1)	139 (19.7)
2–5 weeks	95 (13.5)	106 (15.1)
6–11 weeks	57 (8.1)	46 (6.5)
12 weeks or more	233 (33.1)	237 (33.7)
Hypothesized moderator variables		
Tobacco dependence^*^		
Low	448 (63.5)	430 (61.1)
Moderate	245 (34.8)	264 (37.5)
High	12 (1.7)	10 (1.4)
Quit motivation (“Are you seriously planning to quit?”)		
Within the next 2 weeks	196 (27.8)	189 (26.9)
Within the next 30 days	180 (25.5)	192 (27.3)
Within the next 3 months	265 (37.6)	258 (36.7)
No	63 (8.9)	63 (9.0)
Missing	1 (0.1)	2 (0.3)

Data are *n* (%) unless specified.

^*^Based on heaviness of smoking index (HSI), calculated from baseline number of daily cigarettes and time from waking to first cigarette: low dependence if HSI = 0, 1, or 2, moderate dependence if HSI = 3 or 4, high dependence if HSI = 5 or 6.

### Smoking Outcomes

Table 2 shows the effect of MiQuit on eight smoking outcomes for the pooled data (adjusted OR with 95% CIs). There was a significant increase in the probability of making a quit attempt and on most of the self-reported 7-day smoking outcomes (borderline at late pregnancy), including self-reporting abstinence at both follow-ups. There was no significant effect of MiQuit on the prolonged or validated smoking outcomes.

**Table 2.** Analysis of Smoking Outcomes for the Combined MiQuit RCTs

Smoking Outcome	Usual Care (*N* = 705) % (*n*)	MiQuit (*N* = 704) % (*n*)	Total (*N* = 1409) % (*n*)	Unadjusted *p* value*	Adjusted OR (95% CI)**
Self-reported abstinence outcomes					
(i) 7-day abstinence at 4 weeks post-baseline	4.4 (31)	7.4 (52)	5.9 (83)	.017	1.73 (1.10, 2.74)
(ii) 7-day abstinence at late pregnancy	12.3 (87)	15.9 (112)	14.1 (199)	.054	1.34 (0.99, 1.82)
(iii) 7-day abstinence at both 4 weeks post-baseline and late pregnancy	2.8 (20)	5.7 (40)	4.3 (60)	.008	2.06 (1.19, 3.57)
(iv) Prolonged abstinence from 4 weeks post-baseline to late pregnancy	11.4 (80)	12.4 (87)	11.9 (167)	.557	1.10 (0.80, 1.53)
Abstinence outcomes with biochemical validation at late pregnancy					
(v) 7-day abstinence at late pregnancy	5.4 (38)	7.5 (53)	6.5 (91)	.103	1.43 (0.93, 2.20)
(vi) 7-day abstinence at both 4 weeks post-baseline and late pregnancy	1.7 (12)	3.1 (22)	2.4 (34)	.082	1.86 (0.91, 3.79)
(vii) Prolonged abstinence from 4 weeks post-baseline to late pregnancy	3.8 (27)	5.3 (37)	6.5 (64)	.199	1.39 (0.84, 2.31)
Self-reported quit attempts at late pregnancy					
(viii) Made at least one serious (24 h) quit attempt since baseline	73.6 (335)	80.7 (346)	77.0 (681)	.013	1.49 (1.09, 2.05)

Analyses were complete case for the quit attempt outcome; missing data were coded as non-abstinent for all other outcomes (see “sample size and data attrition” for numbers who provided data per outcome).

Prolonged abstinence was defined as no more than five cigarettes in total during that period. All other smoking outcomes were defined as not smoking “even a puff.”

*Unadjusted, from a χ2 test using a two-sided *p* value.

**From a hierarchical logistic regression model, with adjustment for trial as a random intercept. Statistical significance is denoted (*p* < .05) where the 95% CI does not overlap 1.

### Moderator Analyses

Table 3 and 4 show the effect of MiQuit on eight smoking outcomes per baseline tobacco dependence group and per baseline quit motivation group, respectively, for the pooled data (adjusted OR with 95% CIs). Baseline tobacco dependence had little moderating effect on MiQuit effectiveness. For participants with high baseline quit motivation, there was a significant increase in the quit attempt outcome and most of the self-reported 7-day smoking outcomes, but not the prolonged or validated outcomes. For participants with low baseline quit motivation, where quit rates appeared lower, there were no significant effects of MiQuit on smoking outcomes. There were no significant interactions between the treatment arm and hypothesized moderator variables.

**Table 3.** Moderating Effects of Baseline Tobacco Dependence on MiQuit Effectiveness

Smoking Outcome	Usual Care % (*n*)	MiQuit % (*n*)	Usual Care vs. MiQuit OR (95% CI)	Interaction p*
Self-reported abstinence outcomes				
(i**)** 7-day abstinence at 4 weeks post-baseline				
Low dependence Moderate–high dependence	4.5 (20) 4.3 (11)	7.2 (31) 7.7 (21)	1.66 (0.93, 2.96) 1.85 (.88, 3.93)	.820
(ii) 7-day abstinence at late pregnancy				
Low dependence Moderate–high dependence	13.8 (62) 9.7 (25)	17.4 (75) 13.5 (37)	1.31 (0.91, 1.90) 1.45 (0.85, 2.48)	.771
(iii) 7-day abstinence at both 4 weeks post-baseline and late pregnancy				
Low dependence Moderate–high dependence	3.4 (15) 2.0 (5)	5.4 (23) 6.2 (17)	1.63 (0.84, 3.17) 3.33 (1.21, 9.17)	.247
(iv) Prolonged abstinence from 4 weeks post-baseline to late pregnancy				
Low dependence Moderate–high dependence	12.5 (56) 9.3 (24)	13.5 (58) 10.6(29)	1.09 (0.74, 1.62) 1.15 (0.65, 2.03)	.880
Abstinence outcomes with biochemical validation at late pregnancy				
(v) 7-day abstinence at late pregnancy				
Low dependence Moderate–high dependence	6.5 (29) 3.5 (9)	8.8 (38) 5.5 (15)	1.40 (0.85, 2.32) 1.60 (0.69, 3.71)	.795
(vi) 7-day abstinence at both 4 weeks post-baseline and late pregnancy				
Low dependence Moderate–high dependence	2.0 (9) 1.2 (3)	3.0 (13) 3.3 (9)	1.52 (0.64, 3.59) 2.88 (0.77, 10.74)	.428
(vii) Prolonged abstinence from 4 weeks post-baseline to late pregnancy				
Low dependence Moderate–high dependence	4.9 (22) 1.2 (5)	5.8 (25) 4.4 (12)	1.20 (0.66, 2.15) 2.31 (0.80, 6.65)	.287
Self-reported quit attempts at late pregnancy				
(viii) Made at least one serious (24 h) quit attempt since baseline				
Low dependence Moderate–high dependence	77.3 (228) 66.9 (107)	84.4 (221) 74.9 (125)	1.58 (1.03, 2.44) 1.47 (0.91, 2.38)	.827

Low dependence = HSI score 0–2 (*N* = 448 UC, *N* = 430 MiQuit); moderate–high dependence = HSI score 3–6 (*N* = 257 UC, *N* = 274 MiQuit).

Analyses were complete case for the quit attempt outcome; missing data were coded as non-abstinent for all other outcomes (see “sample size and data attrition” for numbers who provided data per outcome).

*From a hierarchical logistic regression model, with adjustment for trial as a random intercept. Statistical significance is denoted (*p* < .05) where the 95% CI does not overlap 1.

**Table 4.** Moderating Effects of Baseline Quit Motivation on MiQuit Effectiveness

	Usual Care % (*n*)	MiQuit % (*n*)	Usual Care vs. MiQuit OR (95% CI)	Interaction p*
Self-reported abstinence outcomes				
i. 7-day abstinence at 4 weeks post-baseline				
Low motivation High motivation	3.1 (10) 5.6 (21)	3.1 (10) 11.0 (42)	1.02 (0.42, 2.50) 2.09 (1.21, 3.61)	.178
ii. 7-day abstinence at late pregnancy				
Low motivation High motivation	8.8 (29) 15.4 (58)	10.3 (33) 20.7 (79)	1.18 (0.70, 2.00) 1.43 (0.99, 2.08)	.555
iii. 7-day abstinence at both 4 weeks post-baseline and late pregnancy				
Low motivation High motivation	1.8 (6) 3.7 (14)	2.8 (9) 8.1 (31)	1.55 (0.54, 4.40) 2.29 (1.20, 4.38)	.532
iv. Prolonged abstinence from 4 weeks post-baseline to late pregnancy				
Low motivation High motivation	7.6 (25) 14.6 (55)	7.5 (24) 16.5 (63)	0.97 (0.54, 1.74) 1.17 (0.79, 1.73)	.598
Abstinence outcomes with biochemical validation at late pregnancy				
v. 7-day abstinence at late pregnancy				
Low motivation High motivation	3.1 (10) 7.5 (28)	4.4 (14) 10.2 (39)	1.45 (0.63, 3.31) 1.42 (0.85, 2.36)	.963
vi. 7-day abstinence at both 4 weeks post-baseline and late pregnancy				
Low motivation High motivation	0.0 (0) 3.2 (12)	1.9 (6) 4.2 (16)	not estimable 1.33 (0.62, 2.85)	not estimable
vii. Prolonged abstinence from 4 weeks post-baseline to late pregnancy				
Low motivation High motivation	2.1 (7) 5.3 (20)	2.8 (9) 7.4 (28)	1.32 (0.01, 0.05) 1.41 (0.78, 2.55)	.912
Self-reported quit attempts at late pregnancy				
viii. Made at least one serious (24 h) quit attempt since baseline				
Low motivation High motivation	66.7 (144) 80.3 (191)	72.0 (144) 88.1 (200)	1.29 (0.85, 1.95) 1.82 (1.09, 3.05)	.302

Low motivation = not planning to quit within the next 30 days (*N* = 328 UC, *N* = 321 MiQuit); high motivation = planning to quit within the next 30 days (*N* = 376 UC, *N* = 381 MiQuit).

Analyses were complete case for the quit attempt outcome; missing data were coded as non-abstinent for all other outcomes (see “sample size and data attrition” for numbers who provided data per outcome).

*From a hierarchical logistic regression model, with adjustment for trial as a random intercept. Statistical significance is denoted (*p* < .05) where the 95% CI does not overlap 1.

### Mediator Analyses

#### Between-arm Changes in Hypothesized Mechanisms of Action

Table S1 shows between-arm changes, between baseline and late pregnancy, in three hypothesized smoking belief mechanisms of MiQuit action. Scores for determination to quit reduced among both treatment arms between baseline and late pregnancy (mean change: MiQuit −0.16, UC –0.22; *N* = 849) whereas self-efficacy scores increased (MiQuit 0.2, UC 0.05; *N* = 846) and baby harm belief scores increased (MiQuit 0.16, UC 0.08; *N* = 863). Between-arm differences in score changes were nonsignificant. The MiQuit arm reported using significantly more lapse prevention strategies since baseline (mean 8.6 [SE 0.17]) than did the usual care arm (mean 8.1 [SE 0.17]); *N* = 869, *P* = .030.

#### Mediation of the Intervention Effect

Figure S1 shows a path diagram of our four-mediator model (*N* = 875) with coefficients displayed. Changes in belief variables were correlated (quit determination and self-efficacy *r* = 0.74, determination and harm beliefs *r* = 0.29, self-efficacy and harm beliefs *r* = 0.25; all *P* < .001), but not with lapse prevention strategies. All four potential mediators had a significant direct effect (ie, an effect when controlling for other variables) on our primary outcome, self-reported 7-day smoking abstinence in late pregnancy (OR [95% CI]: change in determination to quit 1.43 [1.14, 1.81] and change in self-efficacy to quit 4.97 [3.81, 6.47] were positively associated with smoking abstinence; change in baby harm beliefs 0.77 [0.62, 0.97] and number of lapse prevention strategies used 0.92 [0.87, 0.98] were negatively associated with abstinence). However, MiQuit had a significant direct effect, which was positive, only on number of lapse prevention strategies used (*β* [95% CI]: 0.52 [0.05, 0.99], and not on the three other potential mediators (*β* [95% CI]: change in determination to quit 0.06 [−0.01, 0.22]; change in self-efficacy to quit 0.15 [−0.01, 0.31]; change in baby harm beliefs 0.07 [−0.07, 0.21]), nor on self-reported 7-day smoking abstinence in late pregnancy (OR [95% CI] 1.4 [0.91, 2.16]). Results were not substantively different in a sensitivity analysis using the validated 7-day abstinence outcome in late pregnancy, except for finding no direct effect of determination to quit on abstinence.

Given the results above, number of lapse prevention strategies could have a mediating effect on MiQuit effectiveness. We therefore modelled number of lapse prevention strategies as a single mediating variable between treatment arm and smoking outcome. In this single-mediator model (*N* = 869), the total effect (*β* [95% CI]) was 0.065 [0.008, 0.121]; there was a small but significant indirect (mediated) effect of number of lapse prevention strategies on the relationship between MiQuit and self-reported 7-day abstinence in late pregnancy, but in a negative direction ie, a suppressive effect (*β* [95% CI]: −0.006 [−0.014, −0.000]). When controlling for number of lapse prevention strategies, there was a small but significant direct effect (positive) of MiQuit on smoking abstinence (0.071 [0.015, 0.126]), suggesting a partial but competitive effect of the mediator. The percentage of the total effect mediated was −0.097 (−0.525, −0.048). Model fit for the single mediator model (AIC = 5572, BIC = 5600) was improved from the four-mediator model (AIC = 12 845, BIC = 12 945; 57% reduction), but not improved from the null (no mediator) model (AIC = 917, BIC = 926).

## Discussion

This pooled analysis of two trials provides some evidence that MiQuit, a pregnancy-specific tailored text messaging smoking cessation programme offered to pregnant women with varying levels of motivation to quit smoking, increases the probability of making a quit attempt and of short-term, self-reported smoking outcomes. There is insufficient evidence that MiQuit increases prolonged or validated quit rates, although all effects were in the anticipated direction. There was some evidence of greater MiQuit effectiveness in participants with higher baseline quit motivation (readiness to quit within the next 30 days vs. beyond). In mediation analyses, all four hypothesized mechanisms of MiQuit action were significant predictors of our primary smoking outcome (short-term, self-reported abstinence at late pregnancy), but only the number of lapse prevention strategies used, and not changes in quitting beliefs, was significantly affected by MiQuit; this was negatively associated with abstinence.

Rates of missingness for trial outcome data are a potential weakness (27% at the 4-week follow-up and 36% at late pregnancy across both trials), and rates of biochemical validation were also suboptimal. For our main analysis, we have assumed that people with missing outcome data are smoking (Russell Standard).^29^ We could not assume that our data were missing at random, and the Russell Standard is considered a gold standard for the measurement of smoking cessation outcomes due to it being thought to provide a more conservative estimate than using a complete case approach. In both trials, missingness was slightly higher in the MiQuit arm, meaning that results are unlikely to be biased in favor of the intervention. However, as the rates of missing data between the two treatment groups diverge, it is likely that other more complex methods may need to be adopted to address non-response.^31^ A simulation study using a range of imputation approaches found that some degree of bias was associated with all imputation methods^32^ and therefore further research is needed regarding how to best address this issue.

Reduced statistical power could explain the relative lack of significant effects of MiQuit on smoking outcomes when we split our sample by baseline tobacco dependence or quit motivation. Interaction analyses between MiQuit and these hypothesized moderators were likely underpowered given the low proportions achieving our smoking outcomes, so we interpret the lack of interaction effects cautiously. However, there was clear evidence of an effect of MiQuit on some smoking outcomes for participants who were motivated to quit smoking within the next 30 days, which is useful information for targeting support where it can be most effective. It is possible that we failed to consider important effect mechanisms in our analyses, as we followed a pre-specified protocol aiming to test key variables important to the theoretical basis of MiQuit. We were also unable to investigate, as potential mediators, variables measured only at baseline, such as readiness to quit smoking and tobacco dependence, and, as outcomes were measured only at two time points, our SEM models may have been overly simplistic. Other potential mediators need to be investigated in future studies.

Study strengths are the large sample size and ecologically valid setting. Participants (around a quarter of those eligible) were recruited from 40 antenatal clinics in England, with MiQuit delivered in addition to usual care, and had a wide range of quitting motivation at baseline, so results may be generalizable to routine UK antenatal care settings. We also investigated a broad range of smoking outcomes, from self-reporting a quit attempt to prolonged, validated abstinence, helping to determine text support’s potential usefulness. Few evaluations of interventions have attempted to determine how they achieve their effects, which is important particularly when interventions are theoretically based. Mediation analyses can help us to refine interventions, focusing on components that target effective mechanisms and eliminating ineffective components; it can also provide insights as to why interventions fail.

Cochrane review evidence suggests that text support is effective for achieving prolonged abstinence among general smokers.^33^ Only one study in the review enrolled pregnant women; this reported a significant effect for self-reported 30‐day abstinence among women recruited through a baby health information texting program.^34^ MiQuit appears less effective for prolonged smoking cessation, although effective for shorter-term outcomes. A likely explanation is lower motivation/readiness to quit smoking among participants recruited to the MiQuit trials, as well as a longer period of abstinence required for MiQuit’s prolonged outcome (around 15–36 weeks’ gestation). Willingness to set a quit date was not a selection criterion for the MiQuit trials, participants needed only to agree to receive cessation information, and MiQuit support is designed to include pregnant smokers currently unmotivated to quit (9% of baseline trial participants were “not seriously planning to quit”). Conversely, most review trials appeared to recruit motivated quitters (eg, via advertisements, health websites).^19^ Our analyses showed that pregnant women who were not ready to quit within 30 days (46% at baseline) were not more likely to make a quit attempt in the MiQuit arm than in the usual care arm. This suggests that MiQuit, if used in isolation, might be best targeted to women ready to set a quit date sooner, and could potentially show effectiveness and cost effectiveness if restricted to this group.^19^ Participants in the two MiQuit trials had baseline demographic characteristics that are often associated with lower success in quitting smoking during pregnancy, eg, high proportions had no post-16 qualifications, were not in their first pregnancy and had a partner who smokes. This indicates that MiQuit demonstrated short-term effectiveness among a group that are likely to experience substantial challenges in achieving abstinence. MiQuit has high delivery fidelity, with 98% of participants who were followed up reporting receiving the text messages^18^ and, among those who received them, 87% reporting reading all messages at least once.^16^

For pregnant smokers with varying levels of quit motivation, text support is likely to be insufficient for achieving sustained quitting, and additional content or components are likely to be required to maintain motivation and address factors leading to relapse (eg, withdrawal symptoms, cravings). Environmental factors, which are difficult to change with behavioral self-help interventions such as MiQuit, also contribute to the challenges of quitting smoking in pregnancy, such as partners and peers who smoke, the broader social environment, and socioeconomic factors. Interpersonal NHS cessation support is effective but costly and taken up by relatively few pregnant smokers; however, NRT increases quitting when properly adhered to^35^ and might feasibly be combined with message-based support in a remote delivery model for pregnant smokers unwilling or unable to engage with interpersonal counseling. Additional forms of digital support than texting (eg, self-monitoring apps, online videos, chat functions) might also have the potential for supporting cessation and preventing relapse remotely. Given MiQuit’s potential to increase quit attempts and short-term abstinence through the provision of behavioral support, suggestions for future research are to investigate MiQuit’s potential to boost other cessation interventions when added as an extra component, particularly those that provide NRT, or as part of a multi-component intervention.

An aim of this study was to inform on which determinants of smoking cessation can be effectively targeted by digital support, and which appear most important for driving behavior change. Quitting beliefs were not shown to be significantly affected by MiQuit; determination to quit was high at baseline and reduced by late pregnancy in both arms, while self-efficacy and baby harm beliefs increased more in the MiQuit arm than in the usual care arm but not significantly so. Increases in determination and self-efficacy to quit predicted abstinence in our mediation model (self-efficacy strongly so), suggesting that these are worthwhile targets for behavior change. Number of lapse prevention strategies was slightly higher in the MiQuit arm but by only 0.5 strategies out of a possible fifteen listed, which may not be clinically significant. The slightly negative association between number of lapse prevention strategies used and smoking abstinence is possibly explained by participants trying out more strategies the more they struggle to quit.

The low quit rates typically achieved in smoking cessation trials (eg, for prolonged, validated outcomes) require very large numbers of participants to show between-arm effects. Future evaluations of low-intensity interventions for smoking cessation may wish to consider other, less stringent smoking outcomes, such as the number/proportion of days abstinent since baseline. Digital reporting tools for research participants, such as the NicUse app for reporting daily numbers of cigarettes smoked and other nicotine use,^36,37^ can facilitate data collection for such outcomes. As is typical in trials, MiQuit participants were followed up at discrete time points, and it is possible that periods of smoking abstinence were missed in these and other evaluations.

In conclusion, pregnant women with varying levels of smoking cessation motivation were more likely to report quit attempts and short-term abstinence with the MiQuit intervention but not prolonged, validated abstinence between 4 weeks post-baseline (average 15 weeks’ gestation) and late pregnancy (around 36 weeks’ gestation). It is currently unclear how MiQuit achieves its effects, although increases in quit determination and self-efficacy predicted cessation in our sample. Smoking in pregnancy has a large public health impact and it is imperative that efforts continue to provide effective and appealing quit support.

## Supplementary material

Supplementary material is available at *Nicotine and Tobacco Research* online.

ntae026_suppl_Supplementary_Table_S1

ntae026_suppl_Supplementary_Figure_S1

## Data Availability

Anonymized data is available upon reasonable request.
