# Supplementary material for: The Effectiveness of Text Support for Stopping Smoking in Pregnancy (MiQuit): Multi-Trial Pooled Analysis Investigating Effect Moderators and Mechanisms of Action
Source: Nicotine Tob Res. 2024 Feb 14;26(8):1072–80. doi: 10.1093/ntr/ntae026 (PMC11260894; doi:10.1093/ntr/ntae026)
Supplement: ntae026_suppl_Supplementary_Table_S1 [file ntae026_suppl_supplementary_table_s1.docx]

Supplementary Table 1: Between-arm changes, between baseline and late pregnancy, in potential belief mechanisms of action of MiQuit

| **Potential mechanism of MiQuit action** | **Intervention** | | | **Control** | | | ***P* diff*** |
| --- | --- | --- | --- | --- | --- | --- | --- |
|  | **Baseline** | **Late pregnancy** | **Change (95% CI)** | **Baseline** | **Late pregnancy** | **Change (95% CI)** |  |
| Determination to quit (n=849) | 4.04 (.047) | 3.88 (.056) | -.156 (-.272, -.041) | 4.07 (.045) | 3.85 (.059) | -.222 (-.332, -.112) | .502 |
| Self-efficacy to quit (n=846) | 2.66 (.040) | 2.85 (.062) | .195 (.077, .313) | 2.69 (.041) | 2.73 (.061) | .045 (-.070, .159) | .086 |
| Baby harm beliefs  (n=863) | 4.28 (.049) | 4.44 (.047) | .155 (.050, .261) | 4.32 (.046) | 4.40 (.046) | .083 (-.006, .173) | .395 |

Data are mean (SE), unless otherwise specified.

Potential baseline and late-pregnancy score ranges are 1-5 and change scores –4 to 4.

**All analyses were complete case.**

**P* value from hierarchical linear regression model, controlling for baseline score as a fixed effect, with adjustment for study as a random intercept.
