# Supplementary material for: The Effectiveness of Text Support for Stopping Smoking in Pregnancy (MiQuit): Multi-Trial Pooled Analysis Investigating Effect Moderators and Mechanisms of Action
Source: Nicotine Tob Res. 2024 Feb 14;26(8):1072–80. doi: 10.1093/ntr/ntae026 (PMC11260894; doi:10.1093/ntr/ntae026)
Supplement: ntae026_suppl_Supplementary_Figure_S1 [file ntae026_suppl_supplementary_figure_s1.docx]

Supplementary Figure 1: Path Diagram of MiQuit mediation model


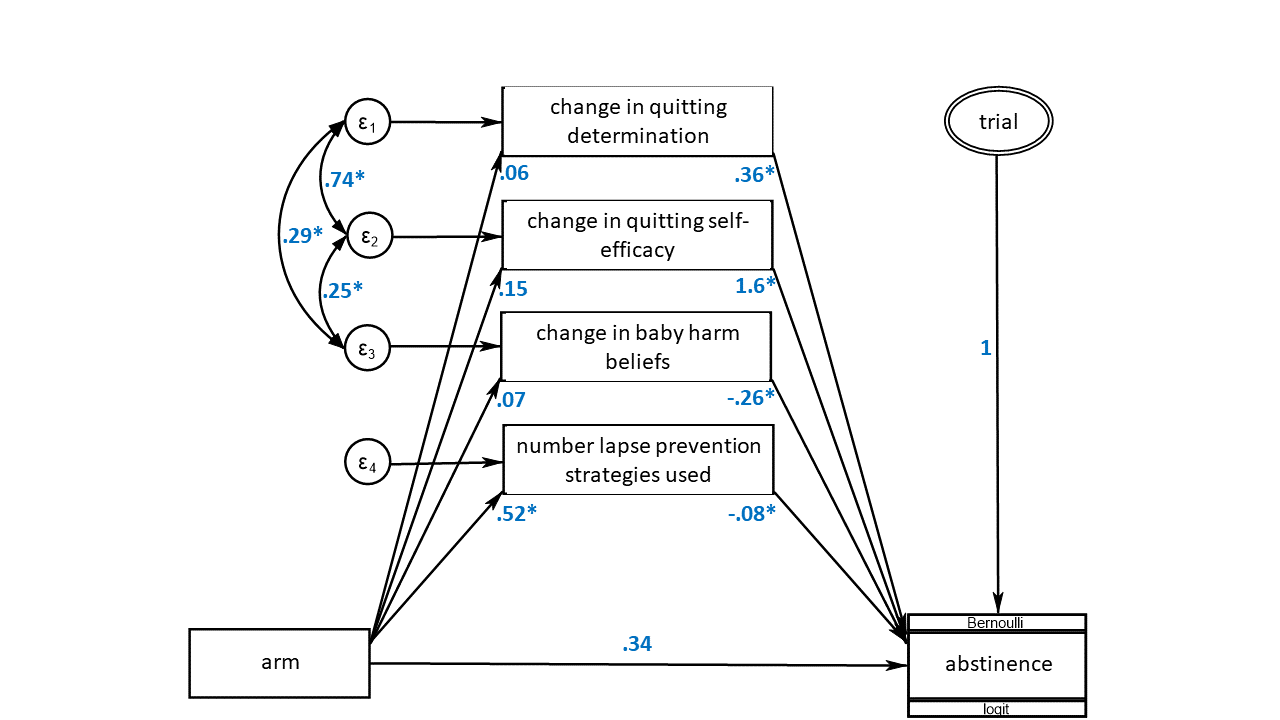


Straight arrows represent causal paths; curved arrows represent correlations.

Trial was modelled as a multilevel variable; all other variables are single-level.

*P<0.05
